# Quantitative analysis of ruminal methanogenic microbial populations in beef cattle divergent in phenotypic residual feed intake (RFI) offered contrasting diets

**DOI:** 10.1186/2049-1891-5-41

**Published:** 2014-08-22

**Authors:** Ciara A Carberry, David A Kenny, Alan K Kelly, Sinéad M Waters

**Affiliations:** 1 Animal and Bioscience Research Department, Animal and Grassland Research and Innovation Centre, Teagasc, Grange, Dunsany, Co. Meath, Ireland; 2 UCD School of Agriculture, Food Science and Veterinary Medicine, College of Life Sciences, University College Dublin, Belfield, Dublin 4, Ireland

**Keywords:** Bovine, qRT-PCR, Residual feed intake, Rumen methaongens

## Abstract

**Background:**

Methane (CH_4_) emissions in cattle are an undesirable end product of rumen methanogenic fermentative activity as they are associated not only with negative environmental impacts but also with reduced host feed efficiency. The aim of this study was to quantify total and specific rumen microbial methanogenic populations in beef cattle divergently selected for residual feed intake (RFI) while offered (i) a low energy high forage (HF) diet followed by (ii) a high energy low forage (LF) diet. Ruminal fluid was collected from 14 high (H) and 14 low (L) RFI animals across both dietary periods. Quantitative real time PCR (qRT-PCR) analysis was conducted to quantify the abundance of total and specific rumen methanogenic microbes. Spearman correlation analysis was used to investigate the association between the relative abundance of methanogens and animal performance, rumen fermentation variables and diet digestibility.

**Results:**

Abundance of methanogens, did not differ between RFI phenotypes. However, relative abundance of total and specific methanogen species was affected (*P* < 0.05) by diet type, with greater abundance observed while animals were offered the LF compared to the HF diet.

**Conclusions:**

These findings suggest that differences in abundance of specific rumen methanogen species may not contribute to variation in CH_4_ emissions between efficient and inefficient animals, however dietary manipulation can influence the abundance of total and specific methanogen species.

## Background

Enteric methane (CH_4_) emissions from ruminant livestock accounts for approximately 80% of emissions from the agricultural-sector [1]. In the rumen, CH_4_ production (methanogenesis) occurs during microbial fermentation of feed by a specific group of microbes known as archaea called methanogens. The majority of CH_4_ produced in the rumen is eructed and thus released into the atmosphere. Enteric CH_4_ production also represents a significant (2-15%) loss in energy from dietary gross energy intake [2]. Therefore, any reduction in enteric CH_4_ emissions would potentially represent both environmental and economic benefits. In beef cattle production, feed efficiency is defined as the efficiency with which dietary substrate is converted into product (e.g. carcass) and is an important determinant of the profitability of meat production [3]. In recent times residual feed intake (RFI) has become the index of choice for measuring feed efficiency [4]. A number of research groups [5,6] including our own [7], have shown that CH_4_ produced in the rumen has been shown to be negatively associated with host feed efficiency.

Enteric CH_4_ emissions are not only influenced by the quantity of feed consumed by ruminants, but also by its chemical composition [8]. For example forage based diets such as grass are composed of structural carbohydrates such as cellulose and hemicellulose, which produce predominantly acetate and butyrate as fermentation end-products compared to the propionate dominant fermentation patterns from a cereal based diets. Recently, using clone-sequencing and next generation methanogen-specific tag-encoded pyrosequencing, we showed that specific species of archaea, *Methanobrevibacter* spp. are the dominant methanogens in the rumen across contrasting diets, with *Methanobrevibacter smithii* being the most abundant species followed by *Methanobrevibacter ruminantium* and *Methanosphaera stadtmanae*[9]. However, particularly when species are present in low abundance, these technologies are not sufficiently accurate to reliably quantify specific methanogens. Quantitative real-time PCR (qRT-PCR) has become a popular method for estimation of methanogen abundance in the ruminant digestive tract [10]. Therefore the objectives of the present study were to quantify the relative abundance of total methanogens and key species *viz Methanobrevibacter smithii*, *M. ruminantium* and *Methanosphaera stadtmanae* in the ruminal fluid of cattle divergent for RFI offered two contrasting diets: a low energy, high forage diet (HF) and a high energy, low forage diet (LF) respectively. Additionally, correlation analysis was used to assess the association between methanogen abundance and animal performance, diet digestibility and rumen fermentation variables.

## Materials and methods

All procedures involving animals were approved for the use of live animals in experiments by the Animal Research Ethics Committee, University College Dublin, Belfield, Dublin, Ireland, and were licensed by the Irish government Department of Health and Children, in accordance with the Cruelty to Animals Act (Ireland 1897) and European Community Directive 86/609/EC (http://ec.europa.eu/food/fs/aw/aw_legislation/scientific/86-609-eec_en.pdf).

### Animal management

This experiment was conducted as part of a larger study designed to examine the physiological control of energetic efficiency in growing beef heifers [11]. Details of the animal experiment used in this study are as previously described [12]. Dietary ingredients and chemical composition have been previously described in detail [11] and both diets were offered *ad libitum*. Briefly, individual dry matter intake (DMI) and growth were recorded for 86 yearling Limousin × Friesian heifers offered *ad libitum* access to a high energy low forage (LF) concentrate based diet over 112 d. The LF diet consisted of 30% corn silage and 70% pelleted concentrate. All animals were subsequently ranked retrospectively on phenotypic RFI. The 14 heifers with the highest (inefficient; high RFI) and 14 heifers with the lowest (efficient; low RFI) RFI coefficients during the study of Kelly et al. [11] were selected for use in the current study. Following initial selection, all 28 animals were re-allocated to a low energy high forage (HF) grass silage diet and individual feed intake recorded for a 44 d period. Following this 44 d period (Period 1) all animals were turned out to pasture for a 56 d dietary “washout” period. Subsequently, all 28 animals were re-housed and re-allocated to a LF concentrate based diet and individual feed intake was recorded for 35 d (Period 2). Individual feed intake and body weight gain were recorded for a further 84 d and RFI was re-calculated. All 28 animals remained within their respective RFI groups [13]. The experiment was therefore designed to have two factors (i) RFI phenotype and (ii) diet type.

### Rumen sampling and methane measurements

Rumen sampling was performed at the end of both dietary periods. Samples of ruminal fluid were collected using a transesophageal sampling device (FLORA rumen scoop; Guelph, Ontario, Canada) according to manufacturer’s instructions. Subsequently, a 20 mL aliquot was transferred using a pipette and sterilized tip into a separate labeled sterilized container, immediately frozen in liquid nitrogen, and stored at -80°C until processing [12].

Methane measurements recorded during the study of Kelly et al. [11] were utilised in the current study (Additional file 1: Table S1). In brief, daily CH_4_ emissions were determined using a calibrated tracer (sulphur hexafluoride (SF_6_)) technique [14] during the last 5 d of each dietary period.

### DNA extraction from ruminal fluid

A detailed description of the DNA extraction method has been described by Carberry et al. [12]. Total microbial DNA was extracted from the 28 ruminal fluid samples by using a repeated bead beating and column purification method which provides efficient recovery of PCR-quality microbial DNA [12].

### Quantitative polymerase chain reaction (qRT-PCR) assays

The differences between SYBR green and TaqMan qRT-PCR chemistries have been reviewed [15]. As previously published primers were available for quantification of total methanogens [16] and *M. stadtmanae*[17] via SYBR green chemistry, we chose to utilise these in our study (Table 1).

**Table 1:** PCR primers used for SYBR green qRT-PCR analysis

**Target Taxon**	**Primer**^**1**^		**E**^**2**^	**Product size, bp**	**Reference**
	**Forward**	**Reverse**			
16 s V3^3^	CCTACGGGAGGCAGCAG	ATTACCGCGGCTGCTGG	2.00	194	Muyzer et al., 1993 [18]
Total methanogens	GGATTAGATACCCSGGTAGT	GTTGARTCCAATTAAACCGCA	1.96	173	Hook et al., 2009 [16]
*Methanosphaera stadtmanae*	CTTAACTATAAGAATTGCTGGAG	TTCGTTACTCACCGTCAAGATC	2.01	150	Zhou et al., 2009 [17]

^1^5’→3’.

^2^Efficiency.

^3^Primers used for qRT-PCR normalisation.

The primers were commercially synthesized (Sigma-Aldrich Ireland Ltd. Dublin, Ireland) and end point PCR was conducted for the validation of the specificity of the primers against target species. For the quantification of *M smithii* and *Methanobrevibcater ruminantium*, it was necessary to utilise TaqMan chemistry for these qRT-PCR assays to allow for species specificity. Details of TaqMan primer and probe sets used in this study, to amplify *M smithii* and *M ruminantium* species are listed in Table 2. For the detection of *M smithii*, primers and a specific FAM labelled probe F were utilised which was designed based on previously published sequences [19]. The same primer set was used in conjunction with a FAM labelled probe designed in the current study to target *M ruminantium* species. The probe was of the same length as the *M smithi* probe with the exception of 3 nucleotide changes in the sequence. All primers and probe sets for the TaqMan qRT-PCR assays were generated using the Primer Express algorithm v1.0 from published sequences (National Centre For Biotechnology Information, NCBI: http://www.ncbi.nlm.nih.gov/) and commercially synthesized (Applied Biosystems, Warrington, UK).

**Table 2:** PCR primers and probes used in this study for TaqManqRT-PCR analysis

***Primer/Probe***	**Sequence (5’→3’)**	**Efficiency**	**Product size, bp**	**Reference**
*Msmithii*F	CCGGGTATCTAATCCGGTTC			Dridi et al., 2009 [19]
*Msmithii*R	CTCCCAGGGTAGAGGTGAAA	1.98	123	Dridi et al., 2009 [19]
*Msmithii* probe	CCGTCAGAATCGTTCCAGTCAG			Dridi et al., 2009 [19]
*Mruminantium* F	CCGGGTATCTAATCCGGTTC			Dridi et al., 2009 [19]
*Mruminantium* R	CTCCCAGGGTAGAGGTGAAA	2.02	123	Dridi et al., 2009 [19]
*Mruminantium* probe	CCGTCAGGTTCGTTCCAGTTAG			Current study

DNA was extracted from individual ruminal fluid samples and diluted to a concentration of 100 ng/μL and 1 μL of diluted DNA was used as a template in all PCR reactions. All SYBR PCR amplifications were initially optimised and performed in 0.5 mL tubes in a DNA thermal cycler (Eppendorf Mastercycler®) using the following program: an initial denaturation step of 95°C for 2 min; 30 cycles of 95°C for 30 s, 60°C for 30 s, 72°C for 30 s and a final elongation step of 72°C for 7 min. The PCR reaction solution (50 μL) consisted of 1 × PCR buffer (final concentrations, 16 mmol/L (NH_4_)_2_ SO_4_, 67 mmol/L Tris–HCl [pH 8.8], 0.01% Tween-20 and 1.5 mmol/L MgCl_2_), 10 pmol of each primer, a 200 μmol/L concentration of each deoxynucleoside triphosphate, 100 ng of template DNA, and 5.0 U of *Taq* DNA polymerse (Bioron, Ludwigshafen, Germany) and 40 μL of molecular grade H_2_O. Aliquots of 10 μL PCR products were analysed by electrophoresis on a 2% (w/v) agarose gel to verify the presence and size of the amplicons. Furthermore amplicons were sequenced to confirm their identity. Using BLAST analysis on the NCBI website, all amplicons were confirmed 100% homologous to their target species. Negative controls without template DNA were included in parallel. Specificity of TaqMan assays for the quantification of *M smithii* and *M ruminantium* were verified before quantification of ruminal DNA. Each probe was validated by running a non-target clone standard as a negative control. In addition, a 10 fold dilution series starting from 25 ng/μL of clones obtained from our own library [9] and identified as either *M smithii* or *M ruminantium* were used as standards and run in triplicate to verify the reproducibility of the TaqMan probe assay. These dilutions were used to calculate PCR efficiencies for each assay. DNA from rumen samples was also diluted and used to calculate PCR efficiencies. For all primer sets, PCR efficiencies were equivalent for both clone libraries and DNA from rumen samples and within the acceptable range to be used to perform qRT-PCR analysis.

The qRT-PCR assays were performed on an ABI 7500 Fast Real-Time PCR system using Fast SYBR® Master Mix (Applied Biosystems, Warrington, UK). Optimization of assay conditions was performed for template DNA concentrations. To reduce inhibition total microbial DNA was diluted to 10 ng/uL. All amplified qRT-PCR reactions were carried out in a 96 well plate format. Non-template controls were included on every plate for each assay to allow screening for possible contamination and primer dimer formation. To minimise variation, all samples included in each analysis were derived from the same extracted DNA batch, prepared under the same conditions and samples were run in triplicate.

All SYBR green reaction mixtures were prepared in a total volume of 20 μL. The reaction consisted of 1 μL DNA, 10 μL Fast SYBR Green master mix, 1 μL of forward and reverse primers (10 ng of each) and 8 μL nuclease-free H_2_O. Thermal cycling conditions applied to each assay consisted of an initial *Taq* activation step at 95°C for 15 min followed by 40 cycles of 95°C for 15 s, 60°C for 60 s, followed by an amplicon dissociation stage (95°C for 15 s, 60°C for 1 min, increasing 0.5°C/cycle until 95°C was reached) which confirmed specificity via dissociation curve analysis of PCR end products. Fluorescence detection was also performed at the end of each denaturation and extension step. TaqMan qRT-PCR assays were performed using TaqMan Fast Universal Master Mix (2×) (Applied Biosystems, Warrington, UK) and prepared in a total volume of 20 μL with 1 μL DNA (10 ng/μL), 1 μL 20× TaqMan assay (forward and reverse primer plus probe), 10 μL TaqMan Fast Universal Master Mix (2×) and 8 μL nuclease-free H_2_O. Thermal cycling conditions applied to each TaqMan assay consisted of an initial hold step at 50°C for 2 min and 95°C for 20 s followed by 40 cycles of 95°C for 3 s and 60°C for 30 s.

### qRT-PCR data analysis

Real time PCR amplification efficiencies (*e*) were estimated for all assays using a linear regression of the threshold cycle (Ct) for each dilution versus the log dilution using the formula: *e* = 5^-1/slope^[20] where ‘5’ is the corresponding fold dilution. Efficiencies of the species specific TaqMan probes and primers sets are presented in Tables 1 and 2. Efficiencies for both SYBR green and TaqMan assays ranged from 1.96 to 2.02, close to the theoretical value of 2.0 which is representative of the doubling effect of the target sequence during the qRT-PCR cycle. All primers had PCR efficiencies between 90% and 110% and were therefore acceptable for use in this study.

Inter-plate calibration based on a calibrator sample included on all plates, efficiency correction of the raw cycle threshold (**Ct**) values and results from triplicate PCR reactions for each target species were averaged and the means calculated using the software package GenEx 5.2.1.3 (MultiD Analyses AB, Gothenburg, Sweden). Within GENEX, the stability of the reference bacterial or methanogen 16S rRNA gene was assessed and assured stable across diet and RFI. Abundance of total methanogens were expressed as a proportion of total estimated rumen bacterial 16S rRNA gene as described previously [21,22] according to the equation: relative quantification = 2^-(Ct target-Ct total bacteria)^, where Ct represents threshold cycle. Abundance of specific methanogen species were expressed as a proportion of total estimated methanogen 16S rRNA gene according to the equation: relative quantification = 2^-(Ct target-Ct total methanogens)^.

### Statistical analysis

All data were analysed using Statistical Analysis Systems v9.1 2002 (SAS Institute, Cary, NC, USA). Data were examined for normality and homogeneity of variance by histograms, qqplots and formal statistical tests as part of the UNIVARIATE procedure of SAS. Data that were not normally distributed were transformed by raising the variable to the power of lambda. The appropriate lambda value was obtained by conducting a Box-Cox transformation analysis using the TRANSREG procedure in SAS. Delta Ct values for total Methanogens were transformed using a lambda value of -0.5 while *M. stadtmanae*, *M smithii* and *M ruminantium* were transformed using a lambda value of 0.25. The transformed data were used to calculate *P*-values. However, the corresponding least squares means and standard errors of the non-transformed data are presented in the results for clarity.

A mixed model ANOVA (PROC MIXED) was conducted to determine the effect of RFI phenotype and diet type on the relative abundance of each species of interest. Fixed effects included RFI phenotype (H or l), diet type (LF or HF), and their interaction. The interaction term, if not statistically significant (*P* > 0.10), was subsequently excluded from the final model. In all analyses the individual animal was denoted as the experimental unit and animal was included as a random effect.

The statistical model used: Y = μ + Ri + Dj + (Rj × Dj) + Ak + ϵijk, where μ was the overall mean, R_i_ was the fixed effect of RFI phenotype (_I_ = H to L), D_j_ the fixed effect of diet (_j_ = LF to HK), R_i_ × D_j_ is the interaction between RFI phenotype and diet type, A_k_ the random effect of animal, and ϵijk is the associated error. Differences between treatments were determined by F-tests using Type III sums of squares. The PDIFF command incorporating the Tukey test was applied to evaluate pairwise comparisons between treatment means.

Spearman partial correlation analysis (PROC CORR, SAS), was calculated to examine associations amongst physiological data, rumen fermentation variables [23] and measured relative methanogen microbial abundance values using analysis with RFI and dietary treatment included as fixed effects in the analysis. Differences were considered significant where *P* < 0.05, while tendencies were considered where *P* < 0.10.

## Results

### qRT-PCR quantification of methanogens

The relative abundance of total methanogens, *Methanosphaera stadtmanae*, *M smithii* and *M ruminantium* are presented in Table 3. No RFI phenotype × diet interactions (*P* > 0.05) were observed for any of the methanogens measured, however a tendency (*P* = 0.08) towards an RFI × diet interaction was observed for the total methanogen population. Additionally, no effect of RFI phenotype was detected for the relative abundance of any of the methanogens analysed (*P >* 0.05). However, diet type affected (*P* = 0.03) the relative abundance of the overall methanogen population with a greater relative abundance of total methanogens in cattle offered the LF compared to the HF diet (Table 3). Furthermore, the relative abundance of *M. stadtmanae* and *M. ruminantium* was higher (*P* < 0.001) when animals were offered the LF compared to the HF diet.

**Table 3:** **Effect of phenotypic RFI and diet on ruminalmethanogen populations**^**1**^

**Items**	**RFI**			**Diet**^**3**^			**Significance**^**4**^		
	**H**	**L**	**SED**	**HF**	**LF**	**SED**	**RFI**	**Diet**	**RxD**
Methanogens^1^	0.07	0.08	0.010	0.07	0.09	0.010	0.71	0.01	0.08
*Methanosphaera stadtmanae*^*2*^	0.36	0.37	0.069	0.09	0.64	0.069	0.44	<0.0001	0.68
*M smithii*^*2*^	0.32	0.25	0.057	0.14	0.43	0.057	0.29	<0.0001	0.33
*M ruminantium*^*2*^	0.18	0.29	0.089	0.04	0.43	0.089	0.65	<0.0001	0.92

^1^Methanogens measured as a proportion of total estimated rumen bacterial 16S rDNA, relative quantification = 2^-(Ct target-Ct total bacteria).^

^2^Methanogen spp. measured as a proportion of total estimated rumen methanogen 16S rDNA, relative quantification = 2^-(Ct target-Ct total methanogens).^

^3^Diet = HF = high forage (grass silage), LF = Low forage (maize silage (30):concentrate (70)).

^4^Significance values for transformed data. Back transformed means presented for clarity. R = RFI, D = DIET.

### Correlation analysis

Correlation coefficients for the association between animal performance, rumen fermentation variables and relative methanogen abundance are presented in Table 4. When animals were offered the LF diet, abundance of total methanogens was positively correlated with acetate (r = 0.44, *P* = 0.02) and acetate: propionate (r = 0.42, *P* = 0.03) and negatively correlated with propionate (r = -0.41, *P* = 0.03) and rumen pH (r = -0.49, *P* = 0.01). *Methanosphaera stadtmanae* was negatively correlated with CH_4_ (r = -0.47, *P* < 0.02). In addition, although not statistically significant a tendency towards a positive relationship between total methanogens and dry matter digestibility (DMD) (r = 0.35, *P =* 0.08), organic matter digestibility (OMD) (r = -0.34, *P* = 0.09) and crude protein digestibility (CPD) (r = -0.38, *P* = 0.05) was observed when animals were offered the LF diet. When animals were offered the HF diet a tendency towards a negative relationship between both *M. smithii* and *M. ruminantium* and CH_4_ (r = -0.36, *P* = 0.07) (r = -0.38, *P* = 0.05) was detected.

**Table 4:** **Association between physiological and rumen fermentation variables and relative methanogen abundance in beef heifers divergent for residual feed intake (RFI)**^**1**^

**Items**	**HF Diet**				**LF Diet**			
	**Methanogens**	***M. stadtmanae***	***M. smithii***	***M. ruminantium***	**Methanogens**	***M. stadtmanae***	***M. smithii***	***M. ruminantium***
DMI^2^	-0.30	-0.05	0.14	0.06	0.13	-0.14	0.22	0.08
CH_4_^3^	-0.27	-0.36	-0.36^a^	-0.38^*^	0.13	-0.47**	-0.07	0.03
MLW^4^	-0.19	-0.07	-0.17	-0.15	0.22	0.14	-0.20	-0.06
tVFA^5^	0.13	0.11	0.08	-0.00	0.01	0.00	-0.00	-0.18
acetate	-0.01	0.06	0.11	-0.04	0.44**	0.07	-0.18	-0.14
propionate	0.01	0.00	-0.13	-0.08	-0.41**	0.30	-0.23	0.09
isobutyrate	0.07	-0.05	-0.12	0.06	0.20	-0.15	-0.21	0.08
butyrate	-0.01	0.06	-0.24	-0.16	0.15	0.03	-0.08	-0.19
isovalerate	-0.01	-0.05	0.08	-0.30	-0.12	-0.13	0.17	0.12
valerate	-0.02	-0.06	0.11	-0.05	-0.31	-0.10	0.14	0.18
A:P^6^	-0.02	0.05	0.14	0.07	0.42**	-0.14	0.22	0.08
pH	-0.11	-0.05	0.14	0.07	-0.49**	0.14	0.22	0.08
CH_4_GEI^7^	-0.01	-0.03	-0.15	-0.15	0.03	-0.23	-0.24	-0.05
DMD^8^	0.21	0.16	-0.21	-0.25	0.35^*^	0.14	-0.21	-0.16
OMD^9^	0.22	0.15	-0.20	-0.14	0.34^*^	0.14	-0.22	-0.15
CPD^10^	0.14	0.20	-0.23	-0.24	0.38^*^	0.17	-0.22	-0.17
NDFD^11^	0.05	0.09	-0.18	-0.14	0.20	0.13	-0.24	-0.16
ADFD^12^	0.05	0.09	-0.17	-0.13	0.26	0.13	-0.24	-0.23
GED^13^	0.17	0.14	-0.18	-0.13	0.35	0.16	-0.25	-0.16

^1^Spearman correlation coefficient in boldface are different from zero (*P* < 0.10).

^2^Dry matter intake.

^3^Methane.

^4^Mean live weight.

^5^Total volatile fatty acids.

^6^Acetate:propionate ratio.

^7^Methane energy from gross energy intake.

^8^Dry matter digestibility.

^9^Organic matter digestibility.

^10^Crude protein digestibility.

^11^Neutral detergent fiber digestibility.

^12^Acid detergent fiber digestibility.

^13^Gross energy digestibility.

^*^P < 0.10, ^**^P < 0.05.

## Discussion

Efficiency of feed utilisation is a key economically relevant trait to the beef cattle industry worldwide given that feed typically accounts for the greatest single input cost [24]. Improved feed efficiency is not only linked to increased profitability, but also reduces the environmental burden, with efficient animals producing less nutrient excretion [25] and reduced CH_4_ emissions [5,6]. As CH_4_ is a terminal product of methanogen mediated feed fermentation, recent research has focused on characterising the methanogen population in animals selected for divergent feed efficiency. Studies [17,26,27] have identified both a diet effect and a correlation between host feed efficiency and rumen microbial composition. *Methanobrevibacter* spp. and *Methanosphaera* sp. are consistently identified as dominant methanogenic archaea in the rumen irrespective of geographical location or dietary feeding regime [17,27-31]. Rumen methanogens were previously characterised by our group [9] in cattle divergent for phenotypic RFI and it was reported that *Methanosphaera* and *Methanobrevibacter* OTUs were identified as the most dominant methanogens. Therefore, in our study total methanogens and specific ruminal methanogen populations of *Methanosphaera, M smithii,* and *M rumantium* were selected for relative qRT-PCR analysis to quantitatively assess whether these populations are associated with variance in feed efficiency and/or dietary energy type in cattle.

There is evidence to suggest that efficient L-RFI animals produce less CH_4_ both on a daily basis (g/d) [6,32,33] and as per unit of body weight (g/kg) [23] than their inefficient H-RFI counterparts. However work conducted from our own group examining the effect of phenotypic RFI on CH_4_ emissions from the animals utilised in the present study showed no difference in CH_4_ between animals ranked as either high or low RFI across the two dietary periods [23]. In our study quantitative analysis showed that the total and specific methanogen communities of animals ranked as either H or L-RFI was not different. This is consistent with previous reports when a low energy feed lot diet was offered [17] and also when the diet was switched from a low to a high energy diet [27]. While there is a sparsity of information in the literature with regard to studies exploring the links between enteric CH_4_ emissions and rumen methanogen abundance there is evidence to suggest that CH_4_ emissions may be consistent with the population size of methanogens [34,35]. However, since the microbial inhabitants of the rumen function in symbiosis, the metabolic activity and substrate specificity of both the methanogens and the wider rumen microbial community must also be considered. Indeed a growing number of studies have reported that methanogen abundance does not reflect the amount of enteric CH_4_ emissions [35-37]. Furthermore, no significant relationship between methanogen abundance and CH_4_ production potential in other ecosystems has been reported [38]. Our previous work [9] reported that the rumen appears to harbour a core group of methanogens regardless of host classification for feed efficiency. Therefore, as suggested by the results obtained for total methanogen abundance in our study, the quantity of the total methanogen population may not be indicative of divergence in CH_4_ yield between RFI phenotypes.

While work from our own group has shown moderate within animal repeatability of RFI while maintained on a constant diet type [13] other recent work has shown that the relative ranking of animals for this trait may change when moved from a low to a high energy diet [39]. This highlights the necessity to investigate both RFI and the rumen microbial community of animals divergent for RFI across different diet types. Previous research has shown that the chemical composition of the diet can have a great effect on the overall rumen bacterial composition in animals divergent for RFI [12]. Despite this, previous reports have suggested that no change in total methanogen abundance of either feed efficient or inefficient cattle divergent for RFI occurs when the diet is changed from a low to high energy diet [27]. Furthermore, it has been suggested that diet has a greater effect on the diversity of the methanogen community rather than total methanogen abundance [17,27]. However, in the current study the abundance of total methanogens was found to be affected by the change in dietary substrate offered. While it was surprising that the relative total methanogen abundance was greater when animals were offered the LF compared to the HF diet, this may have arisen due to the nature of the LF diet (30:70 maize silage:concentrate). With the inclusion of 30% forage in this diet hydrogen fermenting bacteria would still have the ability to proliferate, albeit to a lesser extent, thus providing hydrogen for methanogen growth. In addition, on the LF diet, intakes were higher and there was more easily digestible substrate available to support CH_4_ emissions and the growth of methanogens. Indeed, overall the CH_4_ emissions were higher from animals on the LF diet [23]. Furthermore, decreased rumen pH is often associated with high concentrate diets due to a lowering of the acetate:propionate ratio [40] Decreased ruminal pH can indirectly affect CH_4_ synthesis due to its influence on VFA production [5] and directly through inhibition of methanogen activity [41]. However, data from our own laboratory generated from the same pool of animals as the current study showed that although mean ruminal pH for both high and low RFI phenotypes was lower on the LF diet, the pH did not drop below the optimum range for rumen methanogen growth (pH 6.0-7.5) [23]. Therefore, it is hypothesized that methanogen proliferation would not have been inhibited while animals were offered the LF diet in our study. Differences in our findings to that of others could also have arisen due to differences in qRT-PCR quantification methods. Our qRT-PCR results represent the relative abundance of total methanogens to the total bacterial population (relative quantification) while DNA copy number was used to quantify the methanogen population (absolute quantification) in the study by Zhou et al., [27]. While both these methods have been extensively utilised throughout the literature for the quantification of rumen microbiota, it is important to acknowledge that neither method is without its drawbacks [15]. In the current study, quantification using the relative method was favoured due to the implications of quantification of the target in comparison to a standard curve while using absolute quantification [15]. Additionally, host breed genotype has also been shown to influence methanogen diversity in the rumen and therefore could be a contributing factor to the variation across studies [29].

In the present study when animals were offered the LF diet overall methanogen abundance was positively associated with acetate and negatively associated with propionate. It is widely accepted that both acetate and butyrate promote CH_4_ production, while propionate is considered a rival pathway to methanogenesis [42,43], directly competing with methanogens for available hydrogen during formation [5]. In addition, when total methanogens was correlated with protozoa abundance from our previous study [12] there was strong a positive relationship (data not shown). This positive relationship is compounded by the fact that among H_2_ producers, protozoa have a prominent position, which is strengthened by their close physical association with methanogens, which favours H_2_ transfer from one to the other [37].

An effect of diet type on the relative abundance of *M. stadtmanae* was observed in the present study. This result is consistent with the pyrosequencing analysis conducted in our previous study [9] and may be attributed to the difference in the chemical compostion of the diets and also abundance of other rumen microbes. Energy metabolism of *M. stadtmanae* is more restricted than other methanogenic species, growing only on methanol and H_2_[44]. In the rumen large quantities of methanol are produced via the degradation of pectin by protozoa and other anaerobic bacteria [45]. A HF grass silage based diet is a relatively poor source of dietary pectin, while some constituents of concentrate feeds such as citrus and beet pulp are abundant. We previously reported a tendency towards greater relative abundance of rumen protozoa between the LF and HF dietary periods [12]. Due to the restricted energy metabolism of *Methanosphaera*, the dietary effect observed on this methanogenic species may have arisen due to the fermentation products produced by other rumen microbial populations. In addition, the fastidious energy requirements of *Methanosphaera stadtmanae* compared to other methanogens was most likely the causative reason why when animals were offered the LF diet this methanogenic species was negatively correlated with CH_4_.

The effect of diet on the relative abundance of *M smithii* is most likely due to substrate specificity preference for methanogenesis by this methanogen species. Energy metabolism of *M smithii* is less restricted than *M. stadtmanae* in that it can produce CH_4_ via CO_2_-H_2_ and or formate [45]. In the rumen, formate and hydrogen are the fermentative end products of several types of rumen bacteria and protozoa [46]. We have previously reported a greater abundance of *Fibrobacter succinogenes* and *Prevotella*, both of which produce formate as an end product of fermentation [46], in animals offered LF compared with HF diets [12]. This, coupled with the tendency towards greater relative abundance of hydrogen producing rumen protozoa between the LF and HF dietary periods, suggests that the appropriate substrates for *M smithii* were likely to be in abundance while animals were offered the LF diet. In addition, *M smithii* also possesses enzymes which have been shown in other methanogens to facilitate utilisation of methanol and ethanol [44,47], which are products of bacterial fermentation. Therefore abundance of these methanogens is most likely affected by both chemical composition of the diet and the availability of appropriate end products produced from rumen microbial fermentation.

The relative abundance of *M ruminantium* was higher when animals were offered the LF compared to the HF diet. This is in agreement with Zhou et al. [27] who reported a marked shift in the number of *M ruminantium* when the diet was changed from a low energy to a high energy diet, using PCR-DGGE microbial community analysis. Similar to *M smithii*, it is believed that formate may also be an important substrate for methanogenesis by *M ruminantium*[48]. In addition, the genome sequence of this methanogen has revealed an abundance of encoding adhesion proteins [48]. Initial experiments show that some are involved in mediating close assimilations with hydrogen producing bacteria while others synergise association between protozoa and fungi [48]. Although the LF diet offered in the current study would be expected to yield less CH_4_ emissions compared to a HF diet, the genomic niche adaptation of *M ruminantium* may explain the increase in abundance through mutual associations with other rumen microbial populations.

## Conclusion

In conclusion, this study is the first to quantify both total and a panel of key candidate rumen methanogens in cattle divergent for phenotypic RFI across two contrasting diets. Quantification of the methanogen community showed that feed efficiency alone had no significant effect on the abundance of total or specific methanogens at the species level. However, our results extend the findings of others by demonstrating that the type of dietary substrate offered greatly influences the abundance of specific methanogen species and in addition the density of total methanogens may also be affected by changes in the diet. It is concluded that diet alone has a greater influence on the relative abundance of specific methanogens at a species level. Future work focused on designing specific qRT-PCR assays which target these rarer, less studied methanogens will provide further insight into the effect of host feed efficiency and diet on the rumen methanogen population. Furthermore, studies investigating the association between CH_4_ emissions, rumen methanogen abundance and diet digestibility are required to allow the development of more specific nutritional management strategies to reduce CH_4_ emissions without impacting on host health, growth or productivity.

## Competing interests

The authors declare that they have no competing interests.

## Authors’ contributions

DAK and AKK performed the animal study and collected data relating to animal performance and the rumen samples. SMW and CAC conceptualised the study and were responsible for experimental design. SMW and CAC designed the primers and CAC performed all laboratory and real time PCR analysis. DAK and AKK performed statistical analysis. CAC, SMW and DAK participated in the data collection, data analysis and interpretation. CAC drafted the manuscript. All authors approved the final version of the manuscript for publication.

## Supplementary Material

Additional file 1: Table S1 Information of tested cattle. Click here for file

## References

[B1] Steinfeld H Gerber P Wassenaar T Castel V de Haan C Livestock's long shadow: issues and options. Food and agriculture organization of the united nations 2006 http://www.Fao.Org/docrep/010/a0701e/a0701e00.Htm. Accessed December 21, 2011

[B2] Van Nevel CJ Demeyer DI Control of rumen methanogenesis Environ Mon Assess 1996 42 73 97 10.1007/BF00394043 24193494

[B3] Reynolds CK Crompton LA Mills JAN Improving the efficiency of energy utilisation in cattle Anim Prod Sci 2011 51 6 12 10.1071/AN10160

[B4] Crews DH Genetics of feed utilization and national cattle evaluation: a review Genet Mol Res 2005 4 152 165 16110437

[B5] Hegarty RS Reducing rumen methane emissions through elimination of rumen protozoa Austral J Agri Res 1999 50 1321 1327 10.1071/AR99008

[B6] Nkrumah JD Okine EK Mathison GW Schmid K Li C Basarab JA Price MA Wang Z Moore SS Relationships of feedlot feed efficiency, performance, and feeding behavior with metabolic rate, methane production, and energy partitioning in beef cattle J Anim Sci 2006 84 145 153 16361501 10.2527/2006.841145x

[B7] Fitzsimons C Kenny DA Deighton MH Fahey AG McGee M Methane emissions, body composition, and rumen fermentation traits of beef heifers differing in residual feed intake J Anim Sci 2013 91 5789 5800 10.2527/jas.2013-6956 24146149

[B8] Beauchemin KA Kreuzer M O' Mara F McAllister TA Nutritional management for enteric methane abatement: a review Austral J Exp Agri 2008 48 21 27 10.1071/EA07199

[B9] Carberry CA Waters SM Kenny DA Creevey CJ Rumen methanogenic genotypes differ in abundance according to host residual feed intake phenotype and diet type Appl Environ Microbiol 2014 80 586 594 10.1128/AEM.03131-13 24212580 PMC3911112

[B10] McCartney CA Bull ID Waters SM Dewhurst RJ Technical note: comparison of biomarker and molecular biological methods for estimating methanogen abundance J Anim Sci 2013 91 12 5724 5728 10.2527/jas.2013-6513 24146154

[B11] Kelly AK McGee M Crews DH Jr Fahey AG Wylie AR Kenny DA Effect of divergence in residual feed intake on feeding behavior, blood metabolic variables, and body composition traits in growing beef heifers J Anim Sci 2010 88 109 123 10.2527/jas.2009-2196 19820067

[B12] Carberry CA Kenny DA Han S McCabe MS Waters SM Effect of phenotypic residual feed intake and dietary forage content on the rumen microbial community of beef cattle Appl Environ Microbiol 2012 78 14 4949 4958 10.1128/AEM.07759-11 22562991 PMC3416373

[B13] Kelly AK McGee M Crews DH Jr Sweeney T Boland TM Kenny DA Repeatability of feed efficiency, carcass ultrasound, feeding behavior, and blood metabolic variables in finishing heifers divergently selected for residual feed intake J Anim Sci 2010 88 3214 3225 10.2527/jas.2009-2700 20525931

[B14] Johnson K Huyler M Westberg H Lamb B Zimmerman P Measurement of methane emissions from ruminant livestock using a sulfur hexafluoride tracer technique Environ Sci Technol 1994 28 359 362 10.1021/es00051a025 22176184

[B15] Smith CJ Osborn AM Advantages and limitations of quantitative PCR (q-PCR)-based approaches in microbial ecology FEMS Microbiol Ecol 2009 67 6 20 10.1111/j.1574-6941.2008.00629.x 19120456

[B16] Hook SE Wright A Denis G McBride BW Methanogens: methane producers of the rumen and mitigation strategies Archaea 2010 2010 945785 21253540 10.1155/2010/945785 PMC3021854

[B17] Zhou M Hernandez-Sanabria E Guan LL Assessment of the microbial ecology of ruminal methanogens in cattle with different feed efficiencies Appl Environ Microbiol 2009 75 6524 6533 10.1128/AEM.02815-08 19717632 PMC2765141

[B18] Muyzer G de Waal EC Uitterlinden AG Profiling of complex microbial populations by denaturing gradient gel electrophoresis analysis of polymerase chain reaction-amplified genes coding for 16S rRNA Appl Environ Microbiol 1993 59 695 700 7683183 10.1128/aem.59.3.695-700.1993 PMC202176

[B19] Dridi B Henry M El Khechine A Raoult D Drancourt M High prevalence of *Methanobrevibacter smithii* and *Methanosphaera stadtmanae* detected in the human gut using an improved DNA detection protocol PLoS ONE 2009 4 e7063 10.1371/journal.pone.0007063 19759898 PMC2738942

[B20] Pfaffl MW A new mathematical model for relative quantification in real-time RT-PCR Nucleic Acids Res 2001 29 9 e45 10.1093/nar/29.9.e45 11328886 PMC55695

[B21] Chen XL Wang JK Wu YM Liu JX Effects of chemical treatments of rice straw on rumen fermentation characteristics, fibrolytic enzyme activities and populations of liquid- and solid-associated ruminal microbes in vitro Anim Feed Sci Technol 2008 141 1 14 10.1016/j.anifeedsci.2007.04.006

[B22] Guo YQ Liu JX Lu Y Zhu WY Denman SE Mc Sweeney CS Effect of tea saponin on methanogenesis, microbial community structure and expression of *mcra* gene, in cultures of rumen micro-organisms Lett Appl Microbiol 2008 47 421 426 10.1111/j.1472-765X.2008.02459.x 19146532

[B23] McDonnell RP An examination of the effects of divergent phenotypic selection for residual on enteric methane emissions in beef cattle 2008 Ireland: University College Dublin Masters thesis

[B24] Arthur PF Herd RM Efficiency of feed utilization by livestock – implications and benefits of genetic improvement Can J Anim Sci 2005 85 281 290 10.4141/A04-062

[B25] Herd RM Hegarty RS Dicker RW Archer JA Arthur PF Selection for residual feed intake improves feed efficiency in steers on pasture Anim Prod Austral 2002 24 85 88

[B26] Guan LL Nkrumah JD Basarab JA Moore SS Linkage of microbial ecology to phenotype: correlation of rumen microbial ecology to cattle's feed efficiency FEMS Microbiol Lett 2008 288 85 91 10.1111/j.1574-6968.2008.01343.x 18785930

[B27] Zhou M Hernandez-Sanabria E Guan LL Characterization of variation in rumen methanogenic communities under different dietary and host feed efficiency conditions, as determined by PCR-denaturing gradient gel electrophoresis analysis Appl Environ Microbiol 2010 76 3776 3786 10.1128/AEM.00010-10 20418436 PMC2893468

[B28] Jeyanathan J Kirs M Ronimus RS Hoskin SO Janssen PH Methanogen community structure in the rumens of farmed sheep, cattle and red deer fed different diets FEMS Microbiol Ecol 2011 76 311 326 10.1111/j.1574-6941.2011.01056.x 21255054

[B29] King EE Smith RP St-Pierre B Wright ADG Differences in the rumen methanogen populations of lactating jersey and holstein dairy cows under the same diet regimen Appl Environ Microbiol 2011 77 5682 5687 10.1128/AEM.05130-11 21705541 PMC3165256

[B30] Skillman LC Evans PN Strompl C Joblin KN 16 s rDNA directed pcr primers and detection of methanogens in the bovine rumen Lett Appl Microbiol 2006 42 222 228 10.1111/j.1472-765X.2005.01833.x 16478508

[B31] Whitford MF Teather RM Forster RJ Phylogenetic analysis of methanogens from the bovine rumen BMC Microbiol 2001 1 5 10.1186/1471-2180-1-5 11384509 PMC32158

[B32] Muro-Reyes A Gutierrez-Banuelos H Diaz-Garcia LH Gutierrez-Pina FJ Escareno-Sanchez LM Banuelos-Valenzuela R Medina-Flores CA Corral Luna A Potential environmental benefits of residual feed intake as strategy to mitigate methane emissions in sheep J Anim Vet Adv 2011 10 1551 1556

[B33] Hegarty RS Goopy JP Herd RM McCorkell B Cattle selected for lower residual feed intake have reduced daily methane production J Anim Sci 2007 85 1479 1486 10.2527/jas.2006-236 17296777

[B34] Lillis L Boots B Kenny DA Petrie K Boland TM Clipson N Doyle EM The effect of dietary concentrate and soya oil inclusion on microbial diversity in the rumen of cattle J Appl Microbiol 2011 111 1426 1435 10.1111/j.1365-2672.2011.05154.x 21923746

[B35] Liu C Zhu Z Liu Y Guo T Dong H Diversity and abundance of the rumen and fecal methanogens in altay sheep native to xinjiang and the influence of diversity on methane emissions Arch Microbiol 2012 194 353 361 10.1007/s00203-011-0757-y 22038025

[B36] Machmüller A Soliva CR Kreuzer M Effect of coconut oil and defaunation treatment on methanogenesis in sheep Reprod Nut Dev 2003 43 41 55 10.1051/rnd:2003005 12785449

[B37] Morgavi DP Martin C Jouany J-P Ranilla MJ Rumen protozoa and methanogenesis: not a simple cause-effect relationship Brit J Nut 2012 107 388 397 10.1017/S0007114511002935 21762544

[B38] Liu DY Ding WX Jia ZJ Cai ZC Relation between methanogenic archaea and methane production potential in selected natural wetland ecosystems across china Biogeosciences 2011 8 329 338 10.5194/bg-8-329-2011

[B39] Durunna ON Mujibi FD Goonewardene L Okine EK Basarab JA Wang Z Moore SS Feed efficiency differences and reranking in beef steers fed grower and finisher diets J Anim Sci 2011 89 158 167 10.2527/jas.2009-2514 20817856

[B40] Slyter LL Influence of Acidosis on rumen function J Anim Sci 1976 43 910 929 789319 10.2527/jas1976.434910x

[B41] Pitt RE Van Kessel JS Fox DG Pell AN Barry MC Van Soest PJ Prediction of ruminal volatile fatty acids and pH within the net carbohydrate and protein system J Anim Sci 1996 74 226 244 8778104 10.2527/1996.741226x

[B42] Whitelaw FG Eadie JM Bruce LA Shand WJ Methane formation in faunated and ciliate-free cattle and its relationship with rumen volatile fatty acid proportions Brit J Nut 1984 52 261 275 10.1079/BJN19840094 6433970

[B43] Moss AR Jouany JP Newbold J Methane production by ruminants: its contribution to global warming Ann Zootech 2000 49 231 253 10.1051/animres:2000119

[B44] Fricke WF Seedorf H Henne A Krüer M Liesegang H Hedderich R Gottschalk G Thauer RK The genome sequence of methanosphaera stadtmanae reveals why this human intestinal archaeon is restricted to methanol and H_2_ for methane formation and ATP synthesis J Bacteriol 2006 188 642 658 10.1128/JB.188.2.642-658.2006 16385054 PMC1347301

[B45] Samuel BS Hansen EE Manchester JK Coutinho PM Henrissat B Fulton R Latreille P Kim K Wilson RK Gordan JI Genomic and metabolic adaptations of *Methanobrevibacter smithii* to the human gut Proc Natl Acad Sci U S A 2007 104 10643 10648 10.1073/pnas.0704189104 17563350 PMC1890564

[B46] Hobson PN Stewart CS The rumen microbial ecosystem 1997 London, UK: Blackie academic and professional 467 491

[B47] Berk H Thauer RK Function of coenzyme F_420_-dependent NADP reductase in methanogenic archaea containing an NADP-dependent alcohol dehydrogenase Arch Microbiol 1997 168 396 402 10.1007/s002030050514 9325428

[B48] Leahy SC Kelly WJ Altermann E Ronimus RS Yeoman CJ Pacheco DM Li D Kong Z Mc Tavish S Sang C Lambie SC Janseen PH Dey D Attwood GT The genome sequence of the rumen methanogen Methanobrevibacter ruminantium reveals new possibilities for controlling ruminant methane emissions PLoS ONE 2010 5 e8926 10.1371/journal.pone.0008926 20126622 PMC2812497

