# Supplementary material for: Quantitative analysis of ruminal methanogenic microbial populations in beef cattle divergent in phenotypic residual feed intake (RFI) offered contrasting diets
Source: J Anim Sci Biotechnol. 2014 Aug 22;5(1):41. doi: 10.1186/2049-1891-5-41 (PMC4177383; doi:10.1186/2049-1891-5-41)
Supplement: Additional file 1: Table S1 — Information of tested cattle. [file 2049-1891-5-41-S1.docx]

| **Supplementary Table 1.** Information of tested cattle | | | |
| --- | --- | --- | --- |
| Animal id | RFI ranking | HF CH_4_  (g day ^-1^) | LF CH_4_  (g day ^-1^) |
| 224 | H | 159160 | 148940 |
| 274 | H | 142820 | 197760 |
| 328 | H | N/A | 173630 |
| 375 | H | 95490 | 148060 |
| 396 | H | 121160 | 206900 |
| 634 | H | 119640 | N/A |
| 655 | H | 202990 | 202200 |
| 712 | H | 124380 | 159070 |
| 763 | H | 155060 | 199610 |
| 887 | H | 106320 | 230110 |
| 968 | H | 115030 | 180190 |
| 1264 | H | 93600 | 250870 |
| 1453 | H | 151970 | 158560 |
| 1468 | H | 108720 | 210960 |
| 114 | L | 168610 | 176100 |
| 219 | L | 141860 | 161130 |
| 329 | L | 148080 | 200290 |
| 431 | L | 133800 | 174870 |
| 532 | L | 145650 | 197490 |
| 554 | L | 126110 | 145500 |
| 556 | L | 149450 | 210910 |
| 559 | L | 125120 | 252110 |
| 583 | L | 105940 | 247600 |
| 633 | L | 153260 | 236730 |
| 739 | L | 172670 | 229000 |
| 759 | L | 163730 | 223500 |
| 785 | L | 141940 | 215100 |
| 1196 | L | 114320 | 184370 |
